# Delayed diagnosis of intermittent mesenteroaxial volvulus of the stomach by computed tomography: a case report

**DOI:** 10.1186/1752-1947-2-343

**Published:** 2008-11-11

**Authors:** Colin Yi-Loong Woon, Alexander Yaw-Fui Chung, Albert Su-Chong Low, Wai-Keong Wong

**Affiliations:** 1 Department of General Surgery, Singapore General Hospital, 169608, Singapore; 2 Department of Diagnostic Radiology, Singapore General Hospital, 169608, Singapore

## Abstract

**Introduction:**

Gastric volvulus is a rare condition. Presenting acutely, mesenteroaxial gastric volvulus has characteristic symptoms and may be easily detected with upper gastrointestinal contrast studies. In contrast, subacute, intermittent cases present with intermittent vague symptoms from episodic twisting and untwisting. Imaging in these cases is only useful if performed in the symptomatic interval.

**Case presentation:**

We describe a patient with a long history of intermittent chest and epigastric pain. An earlier barium meal was not diagnostic. Diagnosis was finally secured during the current admission by a combination of (1) serum investigations, (2) endoscopy, and finally (3) computed tomography.

**Conclusion:**

Non-specific and misleading symptoms and signs may delay the diagnosis of intermittent, subacute volvulus. Imaging studies performed in the well interval may be non-diagnostic. Elevated creatine kinase and aldolase of a non-cardiac cause and endoscopic findings of ischaemic ulceration and difficulty in negotiating the pylorus may raise the suspicion of gastric volvulus. In this case, abdominal computed tomography with spatial reconstruction was crucial in securing the final diagnosis.

## Introduction

Gastric volvulus is a rare clinical entity first described by Berti in 1866 [1]. When untreated, complete volvulus, or torsion beyond 180°, results in strangulation and closed loop obstruction, which may lead to ischaemia, necrosis and perforation. Mortality rates may be as high as 30–50% [2,3].

It is thus imperative that the diagnosis is secured early in the course of disease, to allow for early surgical intervention. However, with subacute, intermittent cases, the diagnosis is less apparent as imaging studies performed during the well interval are non-diagnostic.

We describe a case of intermittent mesenteroaxial gastric volvulus with a 1-year history of vague symptoms for which a myriad of investigations were non-diagnostic. It was only during the final admission that a combination of serum investigations, endoscopy, and computed tomography (CT) led to the correct diagnosis.

## Case presentation

**Figure 1:**
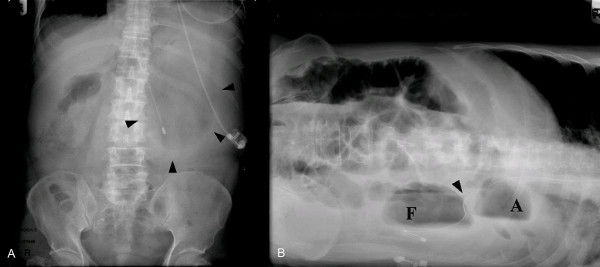
**(A) Supine abdominal radiograph showing a dilated spherical gastric shadow.** (B) Right lateral decubitus abdominal radiograph showing a double gastric bubble, with the superior bubble representing the antrum (A) and inferior bubble being the fundus (F). Nasogastric tube indentation at the cardio-oesophageal junction (arrow) as it enters the stomach.

**Figure 2:**
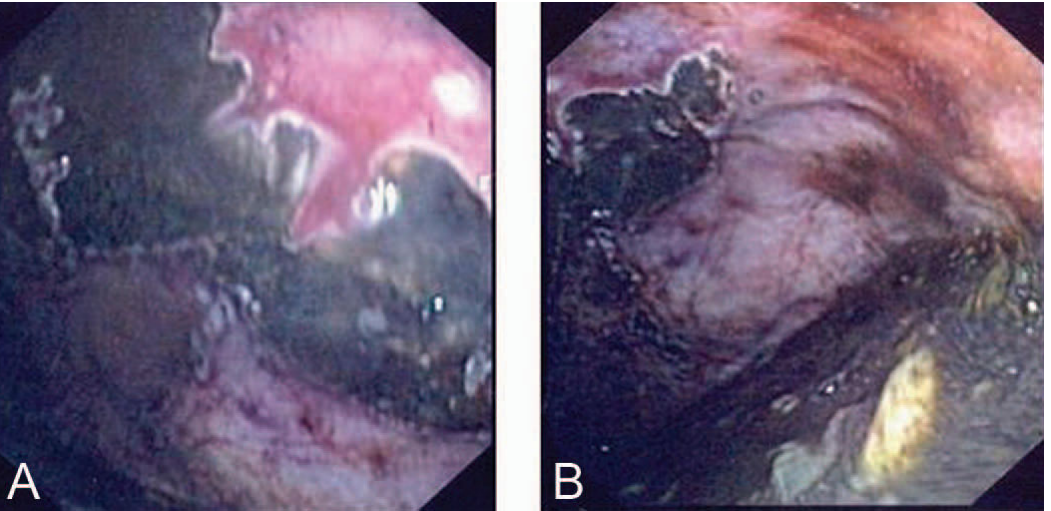
Acute gastric ulcers with surrounding mucosal ischaemia seen on gastroscopy.

**Figure 3:**
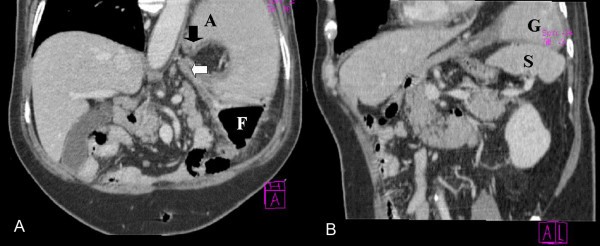
**(A) Coronal reconstructed computed tomography images showing a rotated, 'right-side up' position of the stomach with the pylorus (black arrow) superior to the cardio-oesophageal junction (white arrow).** The fundus (F) is inferior and the antrum (A), superior. (B) Spleen (S) displaced inferior to the gastric body (G).

A 73-year-old male patient had a history of left upper lobectomy for carcinoma of the lung 7 years earlier. He complained of a 1-year history of intermittent atypical chest and epigastric pain for which cardiac investigations were normal and barium meal revealed only gastro-oesophageal reflux. During the current admission, he presented with a 1-day history of epigastric discomfort, nausea and vomiting. Physical examination revealed mild epigastric tenderness. Serum haemoglobin was 14.8 g/dL and total white cell count was 13.6 × 10^9^/litre. Liver function and amylase were normal. Chest radiograph revealed an elevated left hemidiaphragm. Abdominal radiographs revealed an abnormally low position of the presumed site of the cardio-oesophageal junction with an ovoid gastric bubble located in an abnormally low position (Fig. 1). After admission, he developed haematemesis, worsening abdominal pain and increasing tachycardia. Creatine kinase (CK) initially normal, climbed to 2049 U/litre (40–120), despite normal electrocardiogram (ECG) and cardiac troponins. Serum aldolase was elevated at 14.2 U/litre (2–12). Gastroscopy detected acute ischaemic ulceration of the stomach body (Fig. 2) with non-visualization of the pylorus. He was started on proton-pump inhibitors. Follow-up oesophago-gastroduodenoscopy (OGD) was performed twice over 2 weeks, only to reveal similar findings. He reported interval improvement in symptoms, although intermittent low-grade epigastric discomfort persisted. Abdominal CT scan (Fig. 3) performed 19 days after admission finally revealed mesenteroaxial volvulus of the stomach.

**Figure 4:**
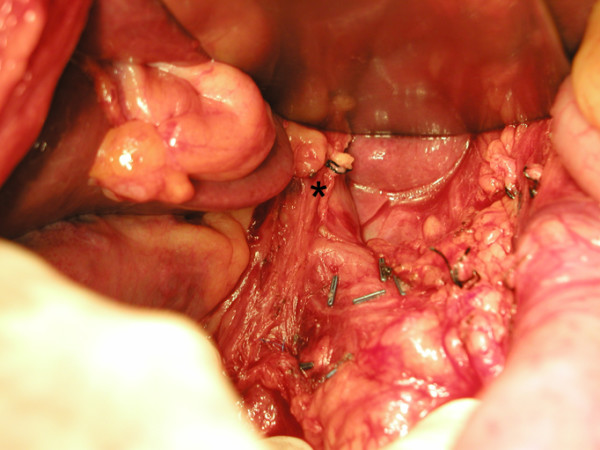
Adhesion band (marked *) between the stomach and the inferior surface of the diaphragm.

**Figure 5:**
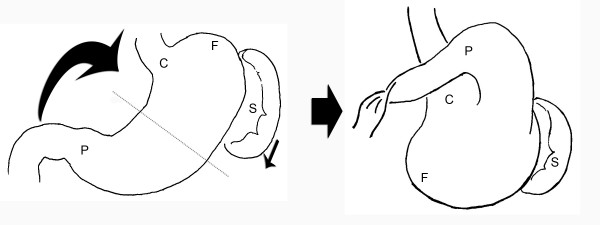
**Diagrammatic representation of mesenteroaxial volvulus.** The axis of rotation is the dotted line that bisects the greater and lesser curves of the stomach. The pyloro-antral region (P) rotates from right to left and anteriorly, with concomitant rotation of the fundus (F) distally, giving the stomach a 'right-side' up view (C, cardia; S, spleen).

**Figure 6:**
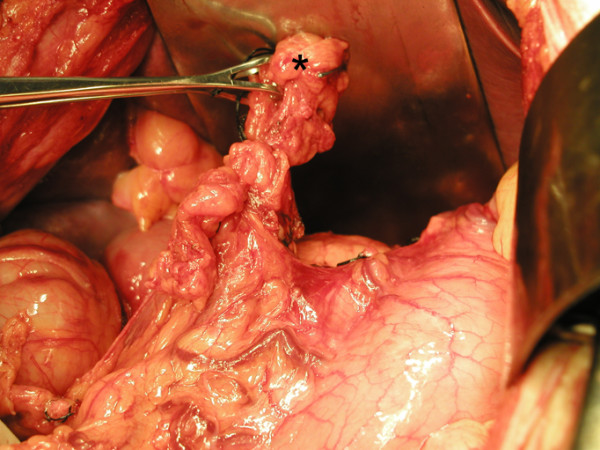
Division of the adhesion band (marked *).

**Figure 7:**
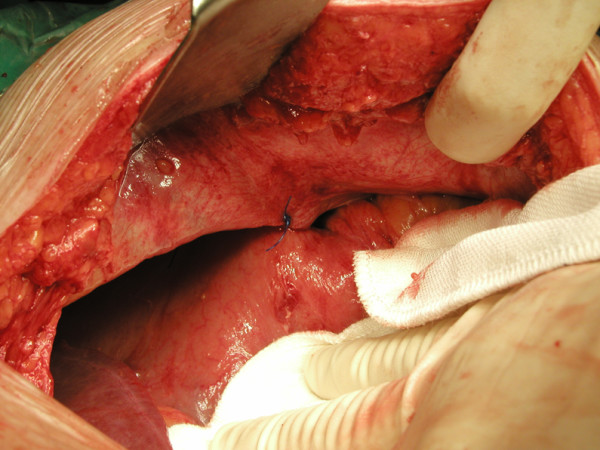
Anterior gastropexy.

At laparotomy the next day, rotation of the proximal two-thirds of the stomach around an adhesion band between the diaphragm and stomach was noted (Fig. 4). This resulted in the pylorus and gastric antrum being pulled up towards the diaphragmatic hiatus (Fig. 5). Otherwise, the stomach was healthy. There was no hiatus hernia or diaphragmatic herniation. The adhesion band was divided (Fig. 6) and anterior gastropexy was performed (Fig. 7). He was discharged well on the 10^th ^postoperative day. Subsequent follow-up over a 1-year period revealed no recurrence of symptoms.

## Discussion

Gastric volvulus has traditionally been classified by the axis of rotation. This patient had Type 2 (mesenteroaxial) volvulus, which occurs in up to 29% of cases. In mesenteroaxial volvulus, the stomach rotates about an axis bisecting the greater and lesser curves. The more common Type 1 (organoaxial) volvulus, occurring in up to 59% of cases, involves rotation of the stomach about an axis connecting the pylorus and cardio-oesophageal junction. The rare Type 3 (2%) is a combination of types 1 and 2 while Type 4 (10%) comprises those that cannot be classified as any of the former [4].

Primary or idiopathic volvulus is less common (30%) [2,5], and is thought to be secondary to laxity of the perigastric (gastrohepatic, gastrosplenic, gastroduodenal and gastrophrenic) ligaments, allowing approximation of cardiac and pyloric ends when the stomach is full, predisposing to volvulus. Secondary volvulus is more common (up to 86% of cases) [2], and is associated with para-oesophageal hiatus hernia, traumatic diaphragmatic hernia, diaphragmatic eventration, previous gastro-oesophageal surgery and other causes of diaphragmatic elevation including phrenic nerve palsy, and intrapleural adhesions [2,3]. In our patient, a raised left hemidiaphragm and a peritoneal adhesion band of unknown aetiology were contributory.

Presenting acutely, gastric volvulus may be associated with the clinical triad of sudden, violent epigastric pain, intractable retching without production of vomitus, and the inability to pass a nasogastric tube into the stomach [3]. Abdominal pain, vomiting and upper GI bleeding may also be present [3]. Subacute, intermittent cases in contrast, produce a vague clinical picture and symptoms may include intermittent upper abdominal distension, early satiety, waterbrash, gastroesophageal reflux or intermittent dysphagia [2,6]. Intermittent atypical chest and epigastric pain in our patient suggests an episodic twisting and untwisting mechanism. In cases of supradiaphragmatic gastric volvulus, chest pain and dyspnoea may be accompanied by clinical findings of bowel sounds in the chest. Carter et al. described three additional findings suggestive of gastric volvulus: minimal abdominal findings when the stomach is in the thorax, gas-filled viscus in the lower chest or upper abdomen on chest radiograph, and obstruction at the site of the volvulus on upper GI series [7].

Biochemical tests are not diagnostic, although hyperamylasemia and elevated serum alkaline phosphatase may be present [8]. In our patient, despite the absence of myocardial ischaemia, CK and aldolase were elevated, suggestive of muscle injury, attributed in this case to strangulation ischaemia.

Plain abdominal radiographs of mesenteroaxial volvulus may show a spherical stomach on supine images, and two air-fluid levels on erect images, with the antrum positioned superior to the fundus [3]. A 'beak' at the location of the cardio-oesophageal junction may be noted. In this case, the supine gastric shadow was ovoid while on lateral decubitus projection, there were two gastric air-filled bubbles with a low cardio-oesophageal junction (Fig. 1A, B). In retrospect, these were highly suggestive of mesenteroaxial volvulus. In contrast, in cases of organoaxial volvulus, the stomach is lying horizontally, with only a single air-fluid level and no beak. These findings should be followed up with upper GI contrast studies (using barium or gastrograffin) that are both sensitive and specific if performed with the stomach in the 'twisted' state. It may show an 'upside-down' stomach and illustrate the degree of obstruction. These investigations remain the most common investigation and provided the greatest yield in 84% of contrast studies done [2,6,9]. In this patient, failure to arrive at a correct diagnosis was likely due to the study being performed in the 'untwisted' state during the well interval.

On endoscopy, distortion of gastric anatomy with difficulty intubating the stomach or the pylorus is highly suggestive. Progressive ischaemic ulceration, or mucosal fissuring suggests late stage disease with strangulation of the gastric blood supply [10].

In this case, abdominal CT was performed because (1) an earlier barium meal was non-diagnostic, (2) endoscopy revealed acute ischaemia, and (3) there was rapid deterioration in the clinical picture (haematemesis, worsening abdominal pain, tachycardia) [2]. Multislice CT allows acquisition of thin-section volume data sets with isotropic voxel dimensions, facilitating display of pathology in a plane of the same spatial resolution as the axially acquired images. This imaging tool simultaneously allows (1) rapid diagnosis of gastric volvulus based on a few coronal reconstructed images, (2) detection of the presence or absence of gastric pneumatosis and free gas suggestive of necrosis and perforation, respectively [8], (3) detection of predisposing factors (e.g. dense adhesions, diaphragmatic or hiatal hernias), (4) exclusion of other extra-gastric or vascular causes of gastric ischaemia. As with upper GI contrast studies, CT scans performed in the well interval ('untwisted' state) may miss the diagnosis entirely. In previously reported series of gastric volvulus, abdominal CT was underutilized and contrast studies remained the imaging modality of choice. Nevertheless, this case illustrates the utility of CT imaging in diagnosing intermittent cases with vague symptoms. Nowadays, CT is easily available and should be the diagnostic tool of choice in any suspected gastric volvulus [11].

After attempting nasogastric decompression, emergency surgery is indicated. Nasogastric decompression is usually only possible in mesenteroaxial volvulus, where the cardia remains open [3,8]. Established pillars of management include reduction of the volvulus, assessment of stomach viability, anterior gastropexy or gastrostomy for prevention of recurrence, and the repair of predisposing factors. Non-viable or frankly gangrenous portions may necessitate subtotal or total gastrectomy. In high risk and elderly patients, the laparoscopic approach provides the advantage of a shorter median hospital stay [2,5,12]. Endoscopic reduction may be attempted in poor-risk patients. The gastroscope is used to untwist the stomach, followed by fixation with percutaneous endoscopic gastrostomy (PEG) [9]. PEG placement, however, fails to prevent recurrent volvulus [2].

## Conclusion

Gastric volvulus is rare. Non-specific and misleading symptoms and signs may delay the diagnosis of intermittent, subacute volvulus. Imaging, performed in the well interval, may be non-diagnostic. Endoscopic abnormalities such as ischaemic ulceration and difficulty in negotiating the pylorus, coupled with biochemical abnormalities such as elevated creatine kinase and aldolase of a non-cardiac cause, should raise the suspicion of gastric volvulus. Abdominal CT scan may prove useful. It provides spatial reconstruction of the acquired images and allows choice of treatment based on additional findings suggestive of necrosis, dense adhesions and associated diaphragmatic or hiatal hernias.

## Consent

Written informed consent was obtained from the patient for publication of this case report and accompanying images. A copy of the written consent is available for review by the Editor-in-Chief of this journal.

## Competing interests

The authors declare that they have no competing interests.

## Authors' contributions

CYLW was involved in drafting the manuscript. AYFC, ASCL and WKW participated in rewriting and revising the report for intellectual content. ASCL provided analysis of the plain abdominal radiographs and CT images. All authors read and approved the final manuscript.
